# Point of care diagnostic of hypercoagulability and platelet function in COVID-19 induced acute respiratory distress syndrome: a retrospective observational study

**DOI:** 10.1186/s12959-021-00293-8

**Published:** 2021-06-02

**Authors:** Johannes Herrmann, Quirin Notz, Tobias Schlesinger, Jan Stumpner, Markus Kredel, Magdalena Sitter, Benedikt Schmid, Peter Kranke, Harald Schulze, Patrick Meybohm, Christopher Lotz

**Affiliations:** 1 Department of Anesthesiology and Critical Care, University Hospital Wuerzburg, Oberdürrbacherstr. 6, 97080 Wuerzburg, Germany; 2 Institute of Experimental Biomedicine, Julius-Maximilians-University Wuerzburg, Wuerzburg, Germany

**Keywords:** COVID-19, Acute Respiratory Distress Syndrome, Point of care testing, Thromboelastometry, Impedance aggregometry

## Abstract

**Background:**

Coronavirus disease 2019 (COVID-19) associated coagulopathy (CAC) leads to thromboembolic events in a high number of critically ill COVID-19 patients. However, specific diagnostic or therapeutic algorithms for CAC have not been established. In the current study, we analyzed coagulation abnormalities with point-of-care testing (POCT) and their relation to hemostatic complications in patients suffering from COVID-19 induced Acute Respiratory Distress Syndrome (ARDS). Our hypothesis was that specific diagnostic patterns can be identified in patients with COVID-19 induced ARDS at risk of thromboembolic complications utilizing POCT.

**Methods:**

This is a single-center, retrospective observational study. Longitudinal data from 247 rotational thromboelastometries (Rotem®) and 165 impedance aggregometries (Multiplate®) were analysed in 18 patients consecutively admitted to the ICU with a COVID-19 induced ARDS between March 12th to June 30th, 2020.

**Results:**

Median age was 61 years (IQR: 51–69). Median PaO_2_/FiO_2_ on admission was 122 mmHg (IQR: 87–189), indicating moderate to severe ARDS. Any form of hemostatic complication occurred in 78 % of the patients with deep vein/arm thrombosis in 39 %, pulmonary embolism in 22 %, and major bleeding in 17 %. In Rotem® elevated A10 and maximum clot firmness (MCF) indicated higher clot strength. The delta between EXTEM A10 minus FIBTEM A10 (ΔA10) > 30 mm, depicting the sole platelet-part of clot firmness, was associated with a higher risk of thromboembolic events (OD: 3.7; 95 %CI 1.3–10.3; *p* = 0.02). Multiplate® aggregometry showed hypoactive platelet function. There was no correlation between single Rotem® and Multiplate® parameters at intensive care unit (ICU) admission and thromboembolic or bleeding complications.

**Conclusions:**

Rotem® and Multiplate® results indicate hypercoagulability and hypoactive platelet dysfunction in COVID-19 induced ARDS but were all in all poorly related to hemostatic complications..

## Background

Hemostatic alterations resulting in severe clinical complications have recently been described in coronavirus disease 2019 (COVID-19) induced acute respiratory distress syndrome (ARDS) [1], [2]. Arterial, venous or microvascular thrombi were found in up to 30 % of COVID-19 intensive care unit (ICU) patients [3]. This COVID-19 associated coagulopathy (CAC) likely differs from sepsis induced disseminated intravascular coagulopathy (DIC). While DIC is early on characterised by a thrombogenic as well as bleeding phenotype, in CAC bleeding events are less common. CAC is associated with increased D-dimer and fibrinogen, as well as elevated cytokine levels accompanied by only minor changes in prothrombin time and platelet count. Pathophysiological mechanisms of CAC include an immune-thrombogenic response due to hyperinflammation and concomitant endothelial dysfunction. Little is known about the role of platelets, whereas hyperactive, hypoactive and exhausted platelets have been described [4], [5]. Consumption of coagulation factors, thrombocytopenia and hyperfibrinolysis only appear late in the disease course [6]. Specific anticoagulation algorithms for CAC have not been established and practice patterns vary considerably between prophylactic and therapeutic use of anticoagulants and antithrombotic agents. In line with recommendations of the International Society on Thrombosis and Hemostasis (ISTH) [7] and the American Society of Hematology (ASH) [8], a current review of the Global COVID-19 Thrombosis Collaborative Group recommends parenteral anticoagulation with heparin in hospitalized patients [9], while more than 10 prospective randomized studies are ongoing.

It is likely that platelet reactivity and coagulation abnormalities change during the course of COVID-19 and rapid Point-of-Care testing (POCT) would provide an important tool in order to adjust therapeutic approaches. Thromboelastometry and thromboelastography provide easy to perform bedside tools [10] and their role in DIC from trauma or sepsis is well-established. Thromboelastometry has already been described as a supplementary tool to evaluate and characterize hypercoagulation in COVID-19 [11]. Nevertheless, specific patterns of platelet reactivity or hypercoagulation are not well characterized. In the current study we investigated the use of thromboelastometry and impedance aggregometry in COVID-19 induced ARDS. Our hypothesis was that specific diagnostic patterns can be identified in patients with COVID-19 induced ARDS at risk of thromboembolic complications utilizing POCT.

## Methods

This is a retrospective, observational study conducted at the University Hospital Wuerzburg, including at total of 18 patients consecutively admitted to the ICU with a COVID-19 induced ARDS between March 12th to June 30th, 2020.

All patients had a SARS-CoV2 infection confirmed with real-time reverse transcriptase polymerase chain reaction (RT-PCR) testing based on the recommended World Health Organization standards. The institutional ethic board of the University of Wuerzburg approved the study (63/20). The need for informed consent from individual patients was waived due to the context of sole retrospective chart review within standard care. Routine clinical data including hemostatic complications, laboratory values were recorded using a patient data management system (PDMS) (COPRA6 RM1.0, COPRA System GmbH, Berlin, Germany). Bleeding events were assessed according to definitions by Schulman et *al.* for major bleedings [12] and Kaatz et *al.* for clinically relevant non-major bleedings [13]. Hemorrhage was classified as a major bleeding if at least one of the following criteria was fulfilled: (1) Fatal bleeding, (2) Bleeding in a critical area or organ, such as intracranial, intraspinal, intraocular, retroperitoneal, intra-articular or pericardial, or intramuscular with compartment syndrome, (3) Bleeding causing a fall in hemoglobin level of 20 g/L or more, leading to transfusion of two or more units of whole blood or red cells. In case a bleeding event did not meet these criteria but did fulfill at least one of the following criteria, it was classified as clinically relevant non-major bleeding: (1) Requiring medical intervention by a healthcare professional, (2) Leading to hospitalization or increased level of care, (3) Prompting a face-to-face evaluation. Thromboembolic events were included in the analysis in case they had been diagnosed by standardized ultrasound examinations or CT scans.

A total of 64 blood samples were analyzed on days 1, 4, 7, and 14 resulting in data from 247 rotational thromboelastometries and 165 impedance aggregometries. Blood samples were taken from a preexisting arterial line and the following order was used: The first 2.0 mL blood tube was discarded, followed by drawing a 3.0 mL plastic tube (polypropylene) containing sodium citrate 0.106 M/L (1:10) as anticoagulant (S-Monovette®, Sarstedt, Nümbrecht, Germany) and one 1.6 mL plastic tube (polypropylene) containing 525 antithrombin units/ mL as anticoagulant (S-Monovette®, Sarstedt, Nümbrecht, Germany). To ensure proper mixing of blood with the anticoagulant, three gentle inversions of the tubes were performed. Tubes were stored at room temperature and analyses were performed within 120 min after blood collection.

Thromboelastometries were conducted on a Rotem® delta (Rotem®, Tem Innovations GmbH, Munich, Germany) from citrate tubes. Impedance aggregometries were performed on a Multiplate® analyzer (Roche Diagnostics GmbH, Mannheim, Germany) from hirudin tubes. Rotem® analysis was conducted at 37 °C and comprised out of INTEM, EXTEM, FIBTEM and HEPTEM measuring clotting time (CT), clot formation time (CFT), amplitude 10 and 30 min after CT (A10/A30), maximum clot firmness (MCF), lysis index 30 min, 45 and 60 min after CT (LI30/LI45/LI60), maximum lysis (ML) and alpha-angle (α) respectively. Multiplate® platelet aggregation was detected at room temperature using thrombin receptor activator peptide 6 (TRAP-6) (final concentration: 32 µM), adenosine diphosphate (ADP) (final concentration: 6.5 µM) and arachidonic acid (ASPI) (final concentration: 0.5 µM), respectively.

Data are reported as median and interquartile range (IQR: 25–75 %). The Chi²-Test and Fisher exact test were used to test the association of dichotomous variables (thromboembolic and bleeding events). A p-value < 0.05 was considered as statistically significant. Correlation coefficient was calculated according to Spearman (r_s_). Data were analyzed by Prism 8.4.3 (GraphPad Software, San Diego, CA) for Microsoft Windows.

## Results

### Patient and Clinical Characteristics

Clinical characteristics and laboratory values are shown in Table 1. The majority of patients were male (67 %). Median age was 61 years (IQR: 51–69) and median BMI was 29.3 (IQR: 25.3–34.6). None of the patients suffered from malignant disease or chemotherapy, had prior inherent or pre-existing coagulation defects or platelet dysfunctions and no pregnancies were reported.

**Table 1:** Clinical characterization and laboratory values of ICU patients (*n* = 18)

Parameters	day 1	day 4	day 7	day 14	ICU stay
Age [years]					61 (51–69)
Body mass index [kg/m^2^]					29.3 (25.3–34.6)
Duration of ICU stay [days]					23 (14–34)
Duration of mechanical ventilation [days]					19 (11–30)
90-day survival [%]					72.2
SOFA (0–24)	12 (10–15)	16 (12–18)	15 (10–16)	12 (5–16)	
APACHE II (0–71)	32 (25–36)	34 (26–39)	32 (29–38)	31 (23–36)	
PaO_2_/FiO_2_ [mmHg]	122 (87–189)	102 (89–158)	138 (106–167)	149 (132–198)	
White blood count [10^3^/µl] (5–10)	9.2 (6.6–11.3)	10.7 (7.7–16.1)	11.9 (7.4–16.3)	11.1 (10.0-15.8)	
Red blood count [10^6^/µl] (4–6)	3.58 (3.14–3.81)	3.11 (2.91–3.45)	3.21 (2.91–3.28)	3.02 (2.74–3.30)	
Hemoglobin [g/dl] (female: 12–16, male: 14–18)	10.0 (8.9–11.1)	9.1 (8.3–10.0)	9.0 (8.6–9.2)	9.2 (8.5–9.4)	
Hematocrit [%] (female: 35–47, male: 42–50)	32.4 (28.3–33.8)	29.0 (26.2–31.1)	29.9 (27.2–30.5)	27.5 (26.0-30.1)	
Platelet count [10^3^/µl] (150–450)	211 (131–282)	206 (145–232)	232 (165–340)	261 (181–328)	
Mean platelet volume [fl] (9.7–11.9)	11.0 (10.3–11.8)	11.1 (10.6–11.5)	11.5 (11.2–12.0)	11.8 (11.1–13.1)	
aPTT [sec] (23–36)	39.6 (37.5–46.2)	46.4 (40.2–56.6)	50.6 (32.9–58.2)	42.5 (31.3–54.1)	
PT [sec] (10–12) (*n* = 17)	11.9 (10.1–12.4)	11.9 (10.2–12.2)	11.2 (10.2–12.0)	11.8 (10.5–12.3)	
INR (0,85 − 1,18)	0.99 (0.94–1.06)	1.01 (0.94–1.06)	0.98 (0.93–1.04)	1.04 (0.99–1.08)	
Quick [%] (70–130)	94 (88–112)	94 (86–113)	103 (92–118)	100 (88–108)	
Fibrinogen [g/l] (2.1-4.0)	6.0 (5.5–7.2)	6.5 (5.7–7.5)	6.1 (4.8–8.4)	5.6 (5.0-6.9)	
D-dimer [mg/l] (< 0.5)	4.7 (2.6–7.3)	2.9 (1.7–5.4)	4.5 (2.3–8.6)	7.2 (4.5–11.4)	
AT [%] (75–125 %)	79 (73–90)	91 (79–103)	106 (87–119)	96 (77–108)	
DIC Score (*n* = 17)	3 (3–3)	3 (2–3)	3 (3–3)	3 (3–3)	
IL-6 [pg/ml] (< 7)	224 (127–511)	247 (81–547)	101 (27–212)	74 (37–308)	
C-reactive protein [mg/dl] (< 0.5)	23.3 (16.1–27.7)	25.14 (14.3–32.5)	18.8 (7.7–29.1)	13.4 (7.7–17.5)	
Procalcitonin [ng/ml] (< 0.5)	1.53 (0.72–3.62)	1.85 (0.50–6.27)	1.45 (0.39–3.86)	1.83 (0.27–2.32)	
Creatinine [mg/dl] (< 1.17)	1.08 (0.80–1.85)	1.08 (0.80–1.85)	1.24 (0.81–2.02)	1.06 (0.86–1.58)	
Anti-Xa level [IU/mL]	0.46 (0.13–0.78) (*n* = 2)	0.38 (0.21–0.55) (*n* = 5)	0.46 (0.32–0.60) (*n* = 7)	0.59 (0.37–0.76) (*n* = 4)	

Clinical characterization and laboratory values of ICU patients (total n=18) in course of ICU stay.

Clinical scores and laboratory values at days 1, 4, 7 and 14 after admission to ICU. Values are expressed as median and IQR. Disseminated Intravascular Coagulation (DIC) scores are calculated from platelet count, prothrombin time, fibrinogen and D-dimers according to guidelines of the International Society on Thrombosis and Haemostasis (ISTH) [35]. Units are displayed in square brackets and reference values in round brackets. Abbreviations: *SOFA* Sequential Organ Failure Assessment Score, *APACHE II* Acute Physiology And Chronic Health Evaluation Score II, *PaO*_*2* _oxygen partial pressure, *FiO*_*2* _fraction of inspired oxygen, *aPTT* activated Partial Thromboplastin Time, *PT* Prothrombin Time, *INR* International Normalized Ratio, *AT* Antithrombin, *IL-6* Interleukin 6.

Prior to ICU admission, three patients received non-steroidal anti-inflammatory drugs (NSAID) (two acetylsalicylic acid (ASA) and one ibuprofen) in their long-term medication. One of these patients, as well as additional two patients received direct Factor Xa inhibitors, respectively. During ICU treament, all patients received therapeutic, weight-adjusted anticoagulation with unfractionated heparin (UFH) or low molecular weight heparin (LMWH), respectively. Moreover, 15 patients were co-treated with prophylactic doses of ASA. All patients had critical illness as evident by a Sequential Organ Failure Assessment (SOFA) Score on admission of 12 (IQR: 10–15) and Acute Physiology And Chronic Health Evaluation II (APACHE II) Score of 32 (IQR: 25–35). Vasopressor support was required in all patients and 12 (67 %) needed renal replacement therapy due to acute renal failure. Pulmonary function was severely impaired with a median PaO_2_/FiO_2_ of 122 (IQR: 87–189) mmHg, indicating moderate to severe ARDS. Due to ARDS progression 10 patients (56 %) were treated with veno-venous extracorporeal membrane oxygenation (vv-ECMO).

Thrombotic events and bleeding complications are shown in Table 2. The majority of patients (78 %) suffered from relevant complications with any form of bleeding in 56 % and thromboembolic events in half of the patients. In total, 2.4 events per patient were recorded.

**Table 2:** Clinical signs of hemostatic alterations (*n* = 18)

Parameters	Number of patients affected	Percentage of patients affected (*n* = 18 total)	Number of events total	Arithmetic mean of events per patient
Any form of hemostatic complication No evidence of hemostatic complication	14 4	78 % 22 %	34	2.4
Any form of bleeding event Major bleeding Clinically relevant non-major bleeding	10 3 8	56 % 17 % 44 %	15 3 12	1.5 1.0 1.2
Any form of thromboembolic event Deep vein/arm thrombosis Pulmonary embolism	9 7 4	50 % 39 % 22 %	19 13 6	2.1 1.1 1.5

Clinical signs of hemostatic alterations (total n=18) in course of ICU stay.

Hemostatic complications comprising bleeding events and thromboembolic events during intensive care for COVID-19. Bleeding events were assessed according to definitions by Schulman et *al.* [12] and Kaatz et *al.* [13]. Within the major bleeding group two patients had intracranial bleeding and one patient developed an extensive subcutaneous hematoma after central venous catheter placement. Thromboembolic events were included in the analysis in case they had been diagnosed by standardized ultrasound examinations or CT scans.

### Point-of-Care Testing

**Fig. 1:**
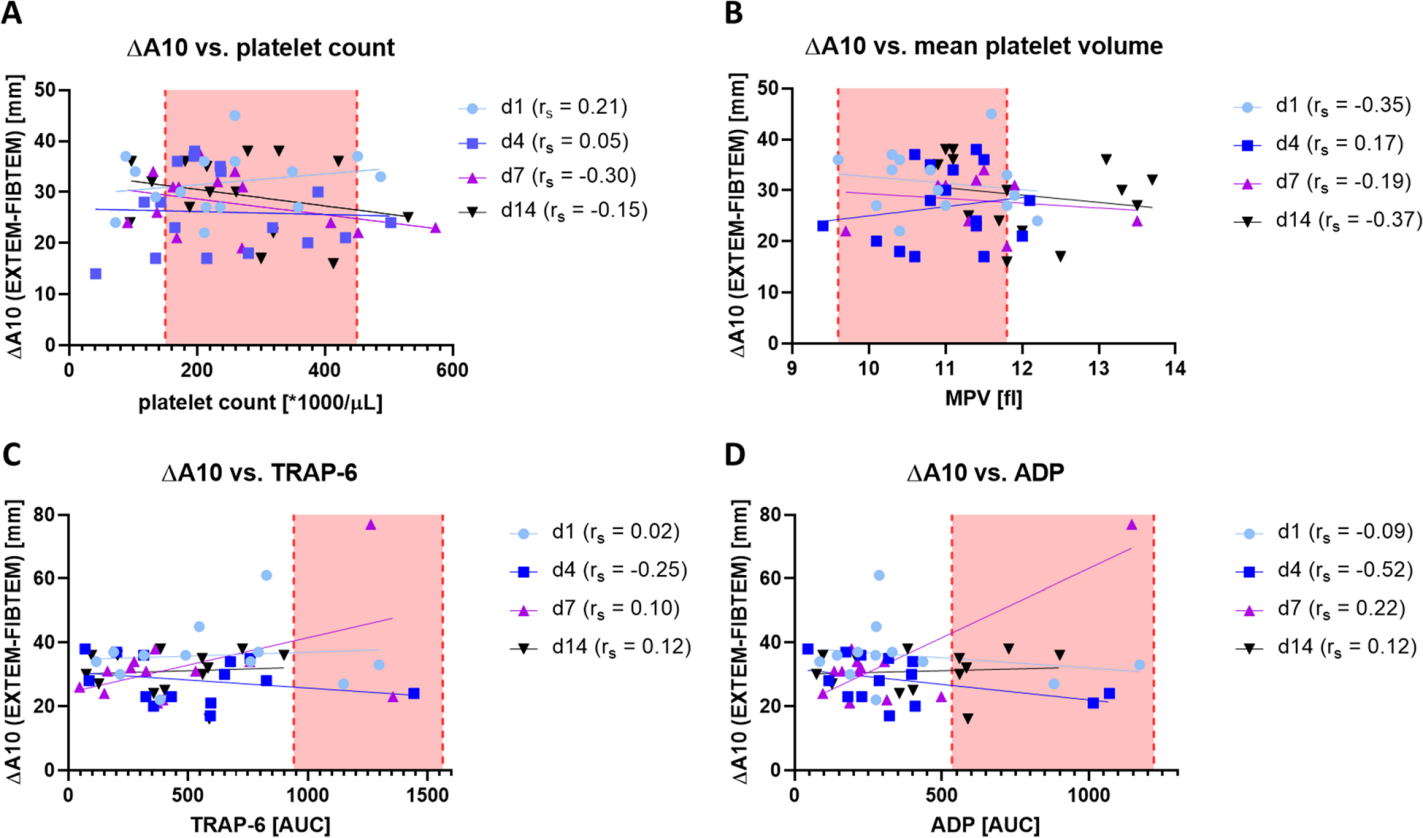
Platelet aggregation ability by Multiplate®. Correlation between ∆A10 (EXTEM minus FIBTEM) and platelet count, mean platelet volume and platelet aggregation ability (Multiplate® with TRAP-6 and ADP). **a** ∆A10 vs. platelet count. **b** ∆A10 vs. mean platelet volume. **c** ∆A10 vs. Multiplate® with agonist TRAP-6. **d** ∆A10 vs. Multiplate® with agonist ADP. Values are presented during the course of ICU stay for day 1 (●), day 4 (■), day 7 (▲) and day 14 (▼) respectively. Spearman correlation coefficient is displayed by r_s_. Reference values for platelet count, mean platelet volume, TRAP-6 and ADP are indicated by pink boxes

**Fig. 2:**
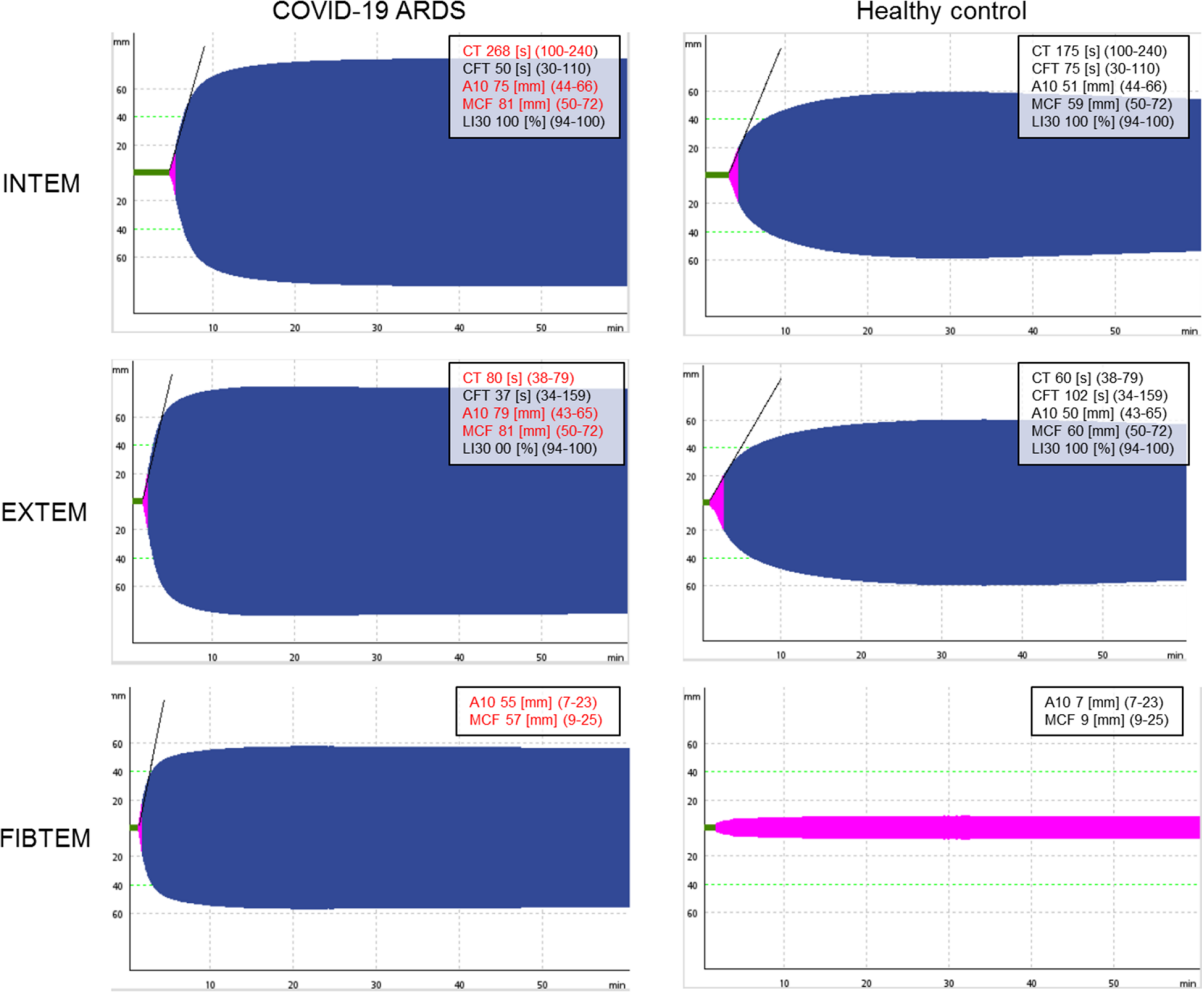
Typical examples of Rotem® INTEM, EXTEM and FIBTEM measurements for a COVID-19 ARDS patient and a healthy control. Typical examples of Rotem® INTEM, EXTEM and FIBTEM measurements for a COVID-19 ARDS patient and a healthy control. Units are displayed in square brackets and reference values in round brackets. Abnormal values are presented in red. Abbreviations: Clotting Time (CT), Clot Formation Time (CFT), Amplitude 10 min after CT (A10), Maximum Clot Firmness (MCF), Lysis Index 30 min (LI30).

Point-of-Care testing by Rotem® and Multiplate® over the course of ICU therapy are depicted in Table 3. Rotem® showed a regular propagation phase of clot formation (i.e. normal CT in INTEM and CFT in INTEM and EXTEM, slightly elevated CFT in EXTEM). LI30 in EXTEM and INTEM were within the normal reference range. We found higher clot strength via elevated A10 and MCF in INTEM, EXTEM and FIBTEM. The sole platelet-part of clot firmness is depicted by the delta between EXTEM minus FIBTEM (expressed as ∆A10 and ∆MCF). ∆A10 and ∆MCF slightly decreased during day 1 to day 4 (∆A10) and day 1 to day 7 (∆MCF). Both values recovered on day 14. At the same time, FIBTEM-A10 and FIBTEM-MCF increased from day 1 to day 7. There was no correlation between platelet count or mean platelet volume and the observed ∆A10 and ∆MCF, respectively (Fig. 1 a and b). Multiplate® point-of-care testing using agonists TRAP-6, ADP and ASPI detected platelet aggregation well below the respective reference ranges (Fig. 1 c and d). There were no significant differences between ECMO and non-ECMO patients for agonists TRAP-6, ADP and ASPI (data not shown). An example of Rotem® measurements in COVID-19 ARDS versus a healthy control is depicted in Fig. 2.

**Table 3:** Rotem® and Multiplate® results in ICU patients (total *n* = 18)

Parameters	day 1	day 4	day 7	day 14
Rotem® INTEM CT [s] (100–240) CFT [s] (30–110) A10 [mm] (44–66) MCF [mm] (50–72) LI30 [%] (94–100)	207 (180–292) 62 (44–75) 70 (61–75) 75 (66–79) 100 (99–100)	273 (206–366) 59 (42–108) 69 (64–75) 75 (72–79) 100 (99–100)	242 (203–297) 45 (41–76) 71 (65–76) 77 (70–79) 100 (100–100)	229 (186–326) 43 (36–59) 72 (67–77) 76 (72–82) 100 (100–100)
Rotem® EXTEM CT [s] (38–79) CFT [s] (34–159) A10 [mm] (43–65) MCF [mm] (50–72) LI30 [%] (94–100)	85 (77–97) 48 (43–59) 70 (62–76) 76 (71–80) 100 (99–100)	87 (73–98) 49 (39–69) 70 (65–75) 76 (72–78) 100 (100–100)	79 (70–85) 39 (35–61) 71 (66–77) 76 (72–80) 100 (100–100)	79 (71–84) 37 (27–56) 72 (67–77) 77 (73–81) 100 (99–100)
Rotem® FIBTEM A10 [mm] (7–23) MCF [mm] (9–25)	38 (31–42) 39 (35–45)	42 (32–52) 46 (36–54)	45 (34–55) 51 (36–61)	43 (31–52) 45 (33–53)
Rotem® Delta ∆A10 (EXTEM minus FIBTEM) [mm] ∆MCF (EXTEM minus FIBTEM) [mm]	33 (27–36) 36 (29–37)	24 (19–35) 29 (22–36)	29 (23–33) 28 (23–35)	30 (24–36) 33 (25–40)
Multiplate® TRAP-6 [AUC] (941–1563) ADP [AUC] (534–1220) ASPI [AUC] (745–1361)	518 (241–819) 277 (155–410) 378 (116–800)	430 (279–676) 287 (181–401) 365 (126–673)	367 (236–744) 214 (154–332) 289 (95–797)	555 (276–809) 402 (166–586) 461 (184–1317)

Rotem® and Multiplate® results at days 1, 4, 7 and 14 after admission to ICU (total *n* = 18).

Point-of-Care testing over the course of ICU treatment. Rotem® results are shown for INTEM, EXTEM, FIBTEM and Delta (EXTEM minus FIBTEM). HEPTEM data are not depicted. Multiplate® results are displayed for the agonists thrombin receptor activator peptide 6 (TRAP-6), adenosine diphosphate (ADP) and arachidonic acid (ASPI) as Area under the Curve (AUC), respectively. Values are expressed as median and IQR. Units are displayed in square brackets and reference values in round brackets. Abbreviations: *CT* Clotting Time, *CFT* Clot Formation Time, *A10* Amplitude 10 min after CT, *MCF* Maximum Clot Firmness, *LI30* Lysis Index 30 min.

There was no correlation between the results of Point-of-Care testing on day 1 and thromboembolic events or bleeding complications. Taking data from the entire course of ICU therapy into account, thromboembolic events were significantly more frequent if ∆A10 exceeded 30 mm (OD: 3.7; 95 %CI 1.3–10.3; *p* = 0.02). Bleeding events were less likely if the EXTEM MCF exceeded the upper reference window (72 mm) (OD 3.9, 95 %CI 1.1–11.9; p = 0.046). LI30 in EXTEM and INTEM was within their reference range.

## Discussion

Severe cases of COVID-19 requiring intensive care are more likely to suffer from CAC, whereas the early identification of patients at risk remains an unresolved question. In the current study, the majority of patients suffered from relevant complications with any form of bleeding in 56 % and thromboembolic events in 50 %. Although the majority of bleeding events was not life threatening, previous publications described a lower incidence of around 5 % [14], [15]. This difference might be caused by a high percentage of ECMO therapy in our study population, which per se is associated with an increased risk of bleeding in COVID-19 [16] and non-COVID-19 patients [17]. The observed high rate of deep vein/arm thrombosis (39 %) and pulmonary embolism (22 %) is actually in line with prior publications reporting venous thrombosis in 27 % [2] and pulmonary embolism in 16.7 % [14] of ICU patients.

Laboratory findings showed a robust increase in D-dimer and fibrinogen levels pinpointing towards the presence of CAC [3]. Platelet counts and mean platelet volumes were almost within their normal range or only slightly increased during the observation period. In large part, the same applied to specific local coagulation tests (International Normalized Ratio (INR), activated partial thromboplastin time (aPTT)), respectively. Mild thrombocytopenia has been frequently observed in patients with COVID-19 and lower nadir platelet counts were associated with increased risk of in-hospital mortality [18]. Meta-analyses indicated that COVID-19 disease progression is associated with lower platelet counts [19] and that thrombocytopenia might be useful for risk stratification [20], [21]. However, the included studies used variable definitions of disease severity. Moreover, elevated platelet counts in severe COVID-19 were also observed [22]. These could be an indicator of a cytokine storm. As such, both an increase and decrease in platelet counts may be a marker of inflammation [23]. Increased fibrinogen levels depict the acute phase response and may also be an important marker of COVID-19 coagulation abnormalities. Elevated fibrinogen levels can be found during initial phase of COVID-19, whereas higher levels are associated with increasing disease severity [24], [14]. As platelets and fibrinogen closely interact in the coagulation cascade, preliminary data indicate that increased mean platelet volumes could increase platelet fibrinogen binding and increased hemostatic potential in severe COVID-19 [25]. Our results in life-threatening illness due to moderate to severe COVID-19 ARDS with high SOFA scores are in accordance with previous publications [6]. Although median platelet counts were within normal range during course of ICU stay, non survivors suffered from more pronounced thrombocytopenia and hyperfibrinogenemia (data not shown).

However, none of the parameters are specific for CAC. In particular increased D-dimers have also been associated with higher incidences of critical illness, thrombosis and acute kidney injury [26].

The primary aim of the current study was to identify and to evaluate the usefulness of POCT as readily available tools for the diagnosis of CAC. Our Rotem® data pointed to a regular propagation phase of clot formation with strong clot firmness and no indication of hyperfibrinolysis during the entire course of ICU stay. These results are in line with prior COVID-19 Rotem® data demonstrating a significantly elevated MCF [11], [27]. Non-COVID-19 studies indicate that such an increased clot firmness could be associated with hypercoagulability [28], [29], [30].

Analysis of platelet function by Multiplate® point-of-care testing using agonists TRAP-6, ADP and ASPI detected a platelet aggregation ability well below the respective reference ranges. Multiplate® analysis could be biased by concomitant application of ASA in 15 patients, as well as a high percentage of ECMO therapy. However, different studies comparing blood from healthy donors and patients with daily ASA intake showed that ASA only reduces platelet aggregation after ASPI challenge, but not after incubation with TRAP-6 [31] or ADP [32]. Therefore, we believe that our Multiplate® results are ASA-independent. ECMO has been shown to reduce platelet aggregation ability after incubation with TRAP-6, ADP and ASPI [33]. We did not find significant differences in platelet aggregation between ECMO and non-ECMO patients. This indicates the presence of a COVID-19 induced, hypoactive platelet dysfunction irrespective of ECMO therapy. These findings differ from other studies in COVID-19 demonstrating increased platelet activation [34], [4]. This might result from the fact that a standardized definition of COVID-19 severity is missing and prior studies defined already severe COVID-19 by the sole requirement of oxygen supplementation.

Aiming to predict clinical events, there was no correlation between the results of POCT on day 1 and thromboembolic events or bleeding complications. During the course of ICU therapy thromboembolic events were more frequent with an elevated ∆A10 > 30 mm. However, as Multiplate® and Rotem® values were not correlated, the contribution of platelets to clot firmness in the majority of patients remains difficult to interpret. Our findings rather indicate that platelets may not predominantly contribute to the increased clot firmness.

Our study has some limitations, which should be carefully considered when interpreting the results. This is a single center study in a highly selected patient population, analyzing data from a low number of patients with life-threatening illness. More than half of these patients received ECMO therapy, which is known to affect different aspects of hemostasis. Moreover, due the retrospective design additional groups with mild COVID-19 could not be included, as Multiplate® and Rotem® POCT was not conducted in non-ICU patients or on a regular basis in non-COVID-19 ARDS patients, respectively.

## Conclusions

In conclusion, monitoring hemostasis and early diagnosis of hemostatic complications in critically ill COVID-19 patients remains challenging. In our patient population of COVID-19-induced ARDS, Rotem® and Multiplate® testing were overall poorly related to hemostatic complications. Nevertheless, our results indicate hypercoagulability and platelet dysfunction, whereas a ∆A10 > 30 mm over the course of therapy may indicate a higher risk of thromboembolic events.

## Data Availability

The datasets used and analysed during the current study are available from the corresponding author on reasonable request.

## Abbreviations

α: Alpha-angle
A10/A30: Amplitude 10 min and 30 min after CT
APACHE II Score: Acute Physiology And Chronic Health Evaluation II Score
ADP: Adenosine diphosphate
ARDS: Acute respiratory distress syndrome
ASA: Acetylsalicylic acid
ASPI: Arachidonic acid
COVID-19: Coronavirus disease 2019
CFT: Clot formation time
CT: Clotting time
ICU: Intensive care unit
LI30/LI45/LI60: Lysis index 30 min, 45 min and 60 min after CT
LMWH: Low molecular weight heparin
MCF: Maximum clot firmness
ML: Maximum lysis
Multiplate®: Impedance aggregometry
NSAID: Non-steroidal anti-inflammatory drugs
PDMS: Patient data management system
POCT: Point-of-care testing
Rotem®: Rotational thromboelastometry
RT-PCR: Real-time reverse transcriptase polymerase chain reaction
SOFA Score: Sequential Organ Failure Assessment Score
TRAP-6: Thrombin receptor activator peptide 6
UFH: Unfractionated heparin
vv-ECMO: Veno-venous extracorporeal membrane oxygenation
